# Antimicrobial Efficacy and Synergy of Metal Ions against *Enterococcus faecium*, *Klebsiella pneumoniae* and *Acinetobacter baumannii* in Planktonic and Biofilm Phenotypes

**DOI:** 10.1038/s41598-017-05976-9

**Published:** 2017-07-19

**Authors:** Misha Y. Vaidya, Andrew J. McBain, Jonathan A. Butler, Craig E. Banks, Kathryn A. Whitehead

**Affiliations:** 1 School of Healthcare Science, Manchester Metropolitan University, Chester St, Manchester M1 5GD UK; 2 School of Health Sciences, Faculty of Biology, Medicine and Health, The University of Manchester, Manchester, M13 9PT UK

## Abstract

The effects of metal ion solutions (silver, copper, platinum, gold and palladium) were determined individually and in combination against *Enterococcus faecium, Acinetobacter baumannii* and *Klebsiella pneumoniae*. Platinum, gold and palladium showed the greatest antimicrobial efficacy in zone of inhibition (ZoI) assays. When tested in combinations using ZoI assays, gold/platinum, gold/palladium and platinum/palladium were indicative of synergy. Microbial inhibitory concentration demonstrated platinum and gold against *Enterococcus faecium*, platinum against *Klebsiella pneumoniae* and platinum and silver against *Acinetobacter baumannii* were optimal. Minimal bactericidal concentrations determined the greatest bactericidal activity was again platinum gold and palladium against all three bacteria. Fractional Inhibitory Concentration (FIC) studies demonstrated that the silver/platinum combination against *Enterococcus faecium*, and silver/copper combination against *Acinetobacter baumannii* demonstrated antimicrobial synergy. Following crystal violet biofilm assays for single metal ion solutions, antimicrobial efficacies were demonstrated for all the metals against all the bacteria Synergistic assays against biofilms demonstrated gold/palladium, gold/platinumand platinum/palladium resulted in the greatest antimicrobial efficacy. Overall, platinum, palladium and gold metal ion solutions in individual use or combination demonstrated the greatest antimicrobial efficacies against planktonic or biofilm bacteria. This work demonstrates the potential for using a range of metal ions, as biocidal formulations against both planktonic or biofilm bacteria.

## Introduction

The antimicrobial properties of metals have been recognised throughout the history of medicine and healthcare^1^. The application of metals in medicine was common until the discovery of antibiotics. Nevertheless, at the beginning of the twenty-first century, a rapid increase in antimicrobial resistance has been observed, which has corresponded with a lack of new antibiotic drugs. To reduce the transmission of potentially infectious microorganisms, there has been a revival of interest in the utilization of metals as antimicrobial/biocidal agents^2^.

There is now a need for alternative biocidal formulations that may be used as active antimicrobials^3, 4^. Biocidal products as outlined in the EU Biocides Directive (98/8/EC), are those that are intended to destroy, render harmless, prevent the action of, or otherwise exert a controlling effect on any harmful organism by chemical or biological means (i.e. disinfectants, antiseptics and preservatives)^2^. Metal surfaces such as silver (Ag) and copper (Cu) have shown antimicrobial efficacies that may control bacterial transmission and infection risks in laboratory settings and hospital environments^5, 6^. Palladium (Pd) alloys have also been considered as potential materials for use as temporary implants to prevent cardiovascular disease infections^7^. Platinum (Pt) possesses metallurgic properties that enable it for use as an antimicrobial in medical implants, such as cardiovascular defibrillators or hip and knee implants and catheters^8^. Gold (Au) nanoparticles have been previously explored for use as delivery vehicles for thioguanine^9^, and various antibiotics^10–12^. The synergistic effects of metals or their complexes have also been demonstrated^13, 14^. For example, phosphogold dithiocarbonate complexes have demonstrated to have comparable antimicrobial potency when compared to various antibiotics such as chloramphenicol against resistant pathogens^13^. An antimicrobial effect has also been demonstrated for Au^+3^ whereby an increase in bacterial inhibition was demonstrated when combined with cephalexin, clindamycin or vancomycin against *Escherichia coli* and *Pseudomonas aeruginosa* ^14^.

*Enterococcus faecium* is an emergent Gram-positive opportunistic pathogen that is the causative agent of several nosocomial infections including complicated urinary tract infections and surgical wound infections^15–17^. *Enterococcus* is difficult to inactivate due to its high level recalcitrance and tolerance of a wide range of growth conditions. *E. faecium* can survive for long periods of time on environmental surfaces including medical equipment, bedrails and door knobs^15, 18, 19^. *Klebsiella pneumoniae* is a Gram-negative microorganism that has a large polysaccharide capsule surrounding the bacterial cell, which both protects the bacteria and acts as a barrier to antimicrobial agents^20^. This species is an opportunistic pathogen principally related with hospital-acquired infections including those of the respiratory and urinary tract^21^. *A. baumannii* also has a role in hospital-acquired infections particularly in causing bacteremia, pneumonia, urinary tract and wound infections^22^. These bacterial strains also have the ability to form biofilms on abiotic and biotic substrates, which may be problematic on catheters, potentially leading to an increase in transmission and infection risks. This may result in increased patient morbidity and mortality^12–14, 23^. In order to determine the potential of a range of metal ions for their use as biocidal agents, a pilot study was carried out to determine the antimicrobial efficacy of five metals, both individually and in combination against *E. faecium, K. pneumoniae and A. baumannii* planktonic or biofilm phenotypes. The potential future use of such antimicrobials would be as biocidal agents for use where intensive cleaning solutions were required.

## Results

### Zone of inhibition (ZoI) for single and combined metals

**Figure 1:**
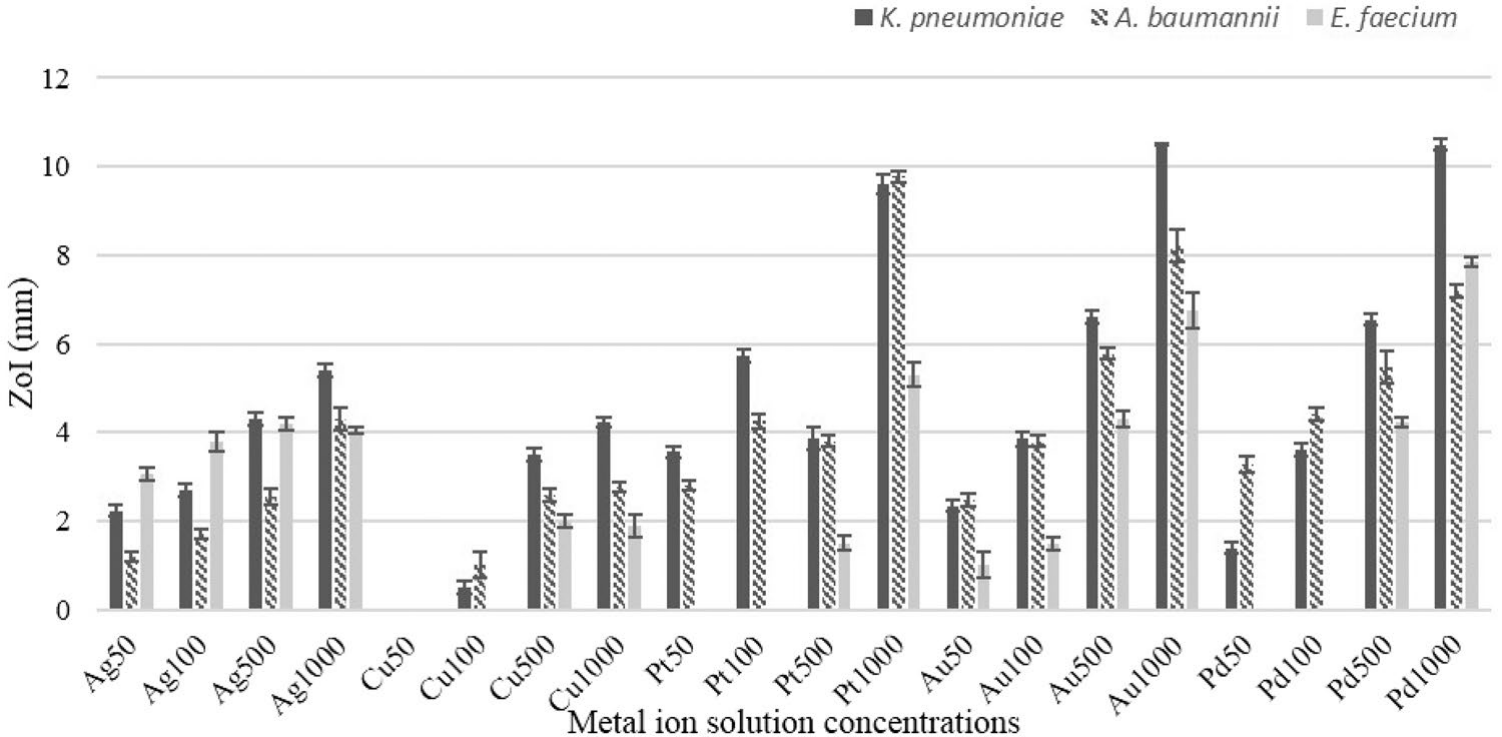
Zone of inhibition values for five metals at different concentrations against tested three pathogens demonstrating that at higher concentrations platinum, gold and palladium were the most effective antimicrobials whereas at lower concentrations silver demonstrated the greatest antimicrobial activity (*p* < 0.001). Au = gold, Cu = copper, Pt = platinum, Pd = palladium and Ag = silver. 50, 100, 500 and 1000 are at concentrations in mgL^−1^ (n = 12).

Zones of inhibition assays were used to determine the antimicrobial efficacies of the metal ions in a semi-solid media. Zone of inhibition assays using the individual or combined metal solutions, demonstrated an increase in antimicrobial activity which correlated with increased metal ion solution concentration (*p* < 0.001) (Fig. 1). When used individually, platinum, gold and palladium demonstrated the greatest ZoIs against all tested microbes (>5 mm and <11 mm) at 1000 mgL^−1^ concentration. Copper demonstrated the lowest antimicrobial efficacies, (>2 mm and <4 mm ZoI) at 1000 mgL^−1^ concentration. Against *E. faecium*, only silver and gold exhibited antimicrobial activity at lower concentrations (50 mgL^−1^) (3 mm and 1 mm respectively).

**Figure 2:**
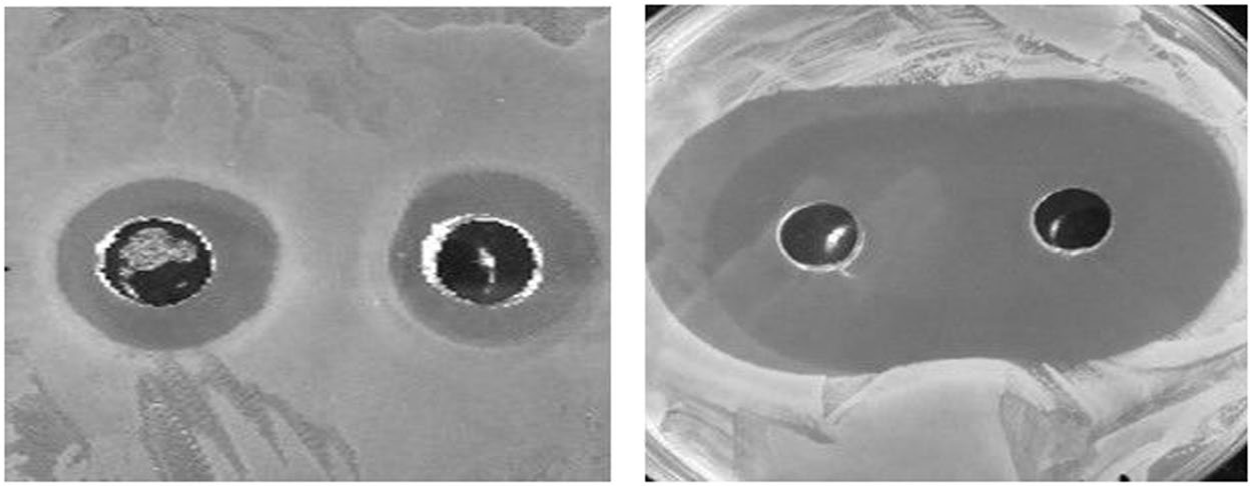
Examples of combined metals used in ZoI to demonstrate the interactions. (**a**) Palladium/platinum against Gram negative bacteria (indifference interaction) and (**b**) gold/palladium against *E. faecium* (synergy interaction).

In order to determine the synergies of the metal ions, they were tested using ZoI assays in combination. It was demonstrated (Table 1), that the metal solutions gold/platinum, gold/palladium and platinum/palladium were most effective antimicrobial combinations and demonstrated synergy at the higher concentrations tested (Fig. 2b). However, most of the metal ion combinations tested using ZoI assays demonstrated an indifferent response (Fig. 2a). Interestingly at the lower concentration of 100 mgL^−1^ silver/platinum, silver/gold and silver/palladium demonstrated antimicrobial activities equivalent to gold/platinum, gold/palladium and platinum/palladium (Table 1).

**Table 1:** Zone of inhibition assays for metal combinations against *E. faecium, A. baumannii and K. pneumoniae* (mm) demonstrating that platinum/palladium, gold/palladium or gold/platinum demonstrated the greatest antimicrobial activity.

		AgCu	AgPt	AgAu	AuPd	CuPt	CuAu	CuPd	AuPt	AuPd	PtPd
50 mgL^−1^	*E. faecium*	0	0	0	0	0	0	0	0	0	0
	*A. baumannii*	1	2	2	2	1	1	1	2	2	2
	*K. pneumoniae*	1	2	2	2	1	1	1	2	2	2
100 mgL^−1^	*E. faecium*	1	2	2	2	1	1	1	2	2	2
	*A. baumannii*	1	2	2	2	1	1	1	4	4	4
	*K. pneumoniae*	1	2	2	2	1	1	1	4	4	4
500 mgL^−1^	*E. faecium*	2	3	3	3	3	3	3	4	4	4
	*A. baumannii*	4	5	5	5	4	4	4	6	6	6
	*K. pneumoniae*	4	5	5	5	4	4	4	6	6	6
1000 mgL^−1^	*E. faecium*	4	4	4	4	4	4	4	6	6	6
	*A. baumannii*	4	6	6	6	6	6	6	8	8	8
	*K. pneumoniae*	4	6	6	6	6	6	6	8	8	8

Au = gold, Cu = copper, Pt = platinum, Pd = palladium and Ag = silver (n = 3). The inhibition zones were graded from 0 to 4, which measured as, 0–4 mm = grade 0, 4–8 mm = grade 1, 8–12 mm = grade 2, 12–16 mm = grade 3 and 16–20 mm = grade 4.

### Minimal inhibitory concentrations (MIC) and minimal bactericidal concentrations (MBC) for single metals

Following the MIC tests, the most effective antimicrobial metal ion solutions were found to be platinum against *K. pneumoniae* (3.90 mgL^−1^), silver and gold (3.90 mgL^−1^) against *A. baumannii* and platinum and gold against *E. faecium* (11.71 mgL^−1^) (Table 2). A similar pattern was demonstrated for the MBC with the greatest bactericidal activity for platinum, gold and palladium at 3.90 mgL^−1^ against *K. pneumoniae*, 5.85 mgL^−1^ (gold) and 7.81 mgL^−1^ (platinum and palladium) against *A. baumannii* and 31.25 mgL^−1^ (gold, platinum and palladium) against *E. faecium*. Silver showed equable antimicrobial efficacy as platinum, gold and palladium against *A. baumannii* (≤7.81 mgL^−1^) and moderate efficacy against rest two pathogens. Copper was found to be the least active with an antimicrobial efficacy at 15.62 mgL^−1^ against the two Gram-negative bacteria and 125.00 mgL^−1^ against *E. faecium* (Table 3).

**Table 2:** Minimal inhibitory concentration and minimal bactericidal concentration (mgL^−1^) values for the metals against tested three bacteria demonstrating that platinum and gold displayed the most inhibitory concentrations and platinum, gold and palladium demonstrated the most potent MBCs.

Test samples	*K. pneumoniae*		*A. baumannii*		*E. faecium*	
	MIC	MBC	MIC	MBC	MIC	MBC
Ag	11.71 ± 2.76	11.71 ± 2.76	3.90 ± 0	7.81 ± 0	15.62 ± 0	62.50 ± 0
Cu	15.62 ± 0	15.62 ± 0	15.62 ± 0	15.62 ± 0	62.50 ± 0	125.00 ± 0
Pt	3.90 ± 0	3.90 ± 0	5.85 ± 1.38	7.81 ± 0	11.71 ± 2.76	31.25 ± 0
Au	5.85 ± 1.38	3.90 ± 0	3.90 ± 0	5.85 ± 1.38	11.71 ± 2.76	31.25 ± 0
Pd	5.85 ± 1.38	3.90 ± 0	7.81 ± 0	7.81 ± 0	15.62 ± 0	31.25 ± 0

Au = gold, Cu = copper, Pt = platinum, Pd = palladium and Ag = silver (n = 3).

**Table 3:** Fractional inhibitory concentration index for metal ion combinations demonstrating a synergistic antimicrobial efficacy for silver/copper against *K. pneumoniae* and silver/palladium against *E. faecium*.

Metal ion combinations	AgCu	AgPt	AgAu	AgPd	CuPt	CuAu	CuPd	AuPt	AuPd	Ptpd
*K. pneumoniae*	0.57 ± 0	0.66 ± 0	0.73 ± 0.68	0.73 ± 0.68	0.92 ± 0.68	0.67 ± 0.68	0.90 ± 0	0.83 ± 0	0.66 ± 0	0.83 ± 0
*A. baumannii*	0.46 ± 0.34	0.61 ± 0.34	0.74 ± 0.34	0.74 ± 0	1.37 ± 1.38	0.62 ± 0	0.73 ± 0	0.83 ± 0	0.74 ± 0	0.57 ± 0
*E. faecium*	0.62 ± 0	1.16 ± 0	0.58 ± 0	0.37 ± 0.68	0.79 ± 0	0.79 ± 0	1.24 ± 0	1.33 ± 0	2.77 ± 0	1.10 ± 0

Synergy = <0.5, indifference = 0.5–4.0 or antagonism = >4.0. Au = gold, Cu = copper, Pt = platinum, Pd = palladium and Ag = silver (n = 3).

### Fractional inhibitory concentration (FIC) for metal combinations

The FIC was used to determine the synergistic antimicrobial efficacy of the metals in combination in a solution. The FIC determined that the silver/palladium combination against *E. faecium* and silver/copper combination against *A. baumannii* demonstrated synergistic antimicrobial efficacy (Table 3) (FIC synergistic value ≤ 0.5). All other metal combinations demonstrated indifferent FIC activities (FIC > 0.5 < 4.0). No metal ion solution combinations were found to demonstrate antagonistic interactions (>4.0) against the three tested microbes (Table 3).

### Biofilm accumulation assays for single and combined metals

**Figure 3:**
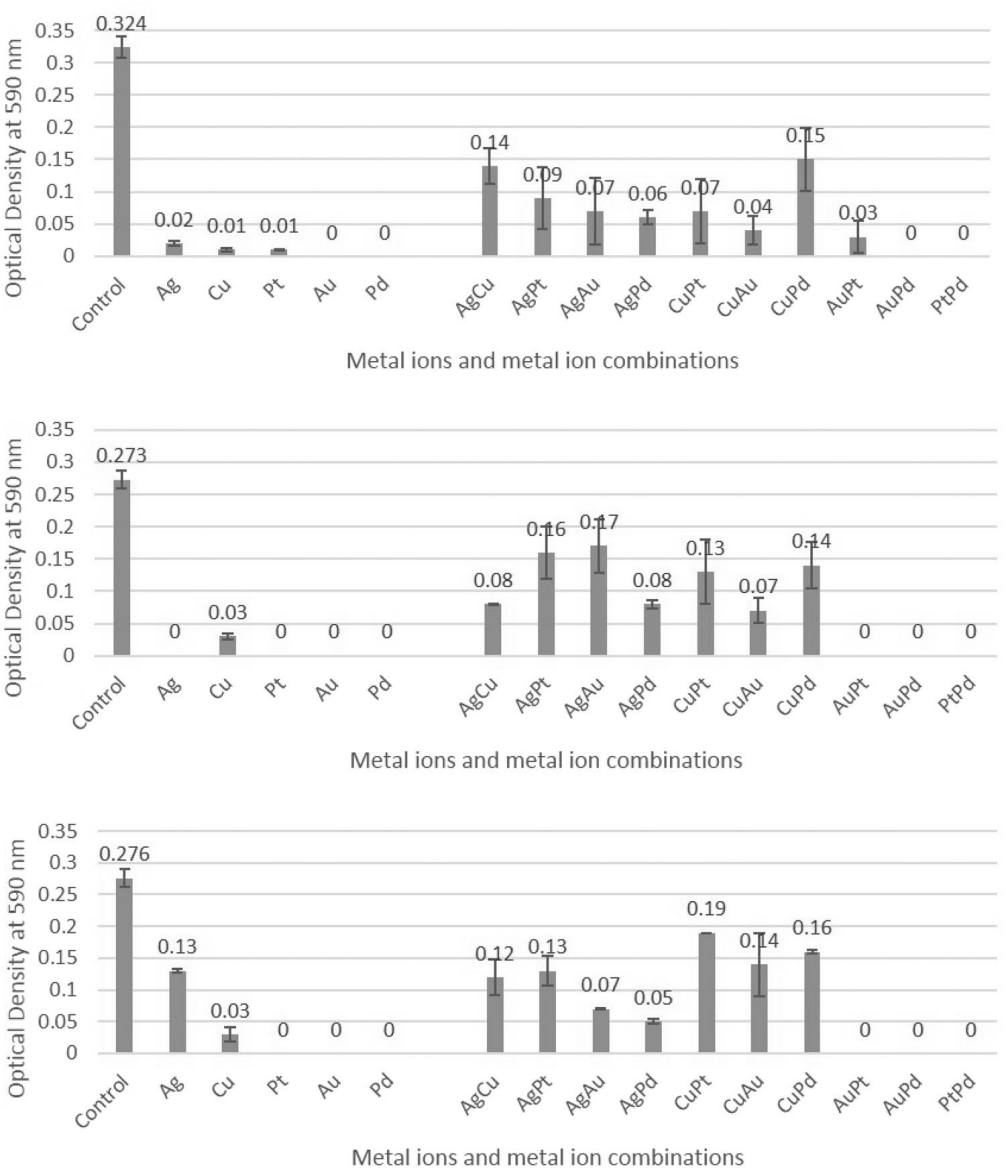
(**a**–**c**) Biofilm growth in the presence of metal ions tested individually and in combination at 500 mgL-1 against (**a**) *K. pneumoniae*, (**b**) *A. baumannii* and (**c**) *E. faecium* using crystal violet biofilm assay. The metals were tested in a 1:1 ratio. Au = gold, Cu = copper, Pt = platinum, Pd = palladium and Ag = silver (n = 3).

The biofilm assays for the individual metal solutions demonstrated excellent antimicrobial efficacies for all the metals at 500 mgL^−1^ concentration against all the three bacteria (Fig. 3a–c). The silver against *E. faecium* was the only result that demonstrated partial antimicrobial activity. Synergistic assays against the biofilms demonstrated that at the greatest concentration (500 mgL^−1^) gold/palladium, gold/platinum and platinum/palladium resulted in the greatest antimicrobial efficacy with no detectable biofilm formation against all three tested bacteria along with silver/palladium and copper/gold against *K. pneumoniae* and silver/palladium against *E. faecium*. Most of the other metal combinations demonstrated partial antimicrobial activity, with only a few demonstrating little antimicrobial activity. None of the metal ion combinations demonstrated no antimicrobial activity.

## Discussion

With the increase in hospital-acquired infections and the development of multidrug resistant bacteria, it is imperative that new biocidal and antimicrobial formulations are found. Metal compounds and complexes of palladium, platinum, copper, gold and silver have been shown to demonstrate effective antimicrobial efficacies against a broad range of AMR pathogens^5, 6, 10, 19^. Metals such as silver in wound dressings, copper on touch surfaces of patient equipment and gold and palladium coatings on catheters are considered to possess potential antimicrobial properties to reduce bacterial infection and transmission risks^24–26^. However, few studies have demonstrated the antimicrobial efficacy of platinum, gold and palladium in their ionic forms or in combination, although their complexes (tetradentate macrocyclic, cisplatin, etc.) have been shown to demonstrate inhibition against bacterial pathogens^27–29^.

As expected, concentration played a pivotal role in increasing the antimicrobial activity in the present study. Similar results have been demonstrated by others for complexes containing N-(thiophen-2-ylmethylene)benzo[*d*]thiazol-2-amine schiff bases with copper, zinc, cobalt and nickel whereby antimicrobial efficacy increased with increasing concentrations (from 5 mgL^−1^ to 20 mgL^−1^)^30^. Copper, nickel and cobalt combined with coumarin-8-yl ligands have also showed greater bacterial inhibition at 100 mgL^−1^ compared to 25 mgL^−1^ or 50 mgL^−1^ against *K. pneumoniae* ^31^. This is probably due to the greater quantity of metal ions resulting in increased metal-bacterial interactions, leading to increased cell death^14^.

Overall, platinum, gold and palladium demonstrated the greatest antimicrobial efficacies against both planktonic bacteria and biofilms. However, in its ionic form, copper demonstrated little antimicrobial effect against cells or biofilms. In our work, which used metal ions in solution, the most electronegative metals produced the best antimicrobial results overall. This may be a result of the high electronegativity of the metal ions being highly attracted to the negatively charged bacteria. The result of these highly attractive forces may result in increased bacterial-metal ion interactions leading to greater antimicrobial efficacy and thus increased cell death.

Although copper and silver are known antimicrobials these were not the most active metal ion solutions tested. The platinum, gold and palladium being the most effective antimicrobials was determined in all the assays, except in the results from the FIC. The FIC demonstrated that the silver combinations (silver/palladium against *E. faecium* and silver/copper against *A. baumannii*), demonstrated the most antimicrobial synergistic combination. This might be since, in our work the metal ions were in solutions whereas work by others usually involves the investigation of the antimicrobial efficacy of the metals in the form of surfaces, nanoparticles or complexes and it is known that the form of the metal will affect the antimicrobial mechanism of action. Although the MIC, MBC and FIC were carried out in liquid media, it may be that when the metals were combined as in the FIC, the metal ions interacted in a different way, resulting in the silver having a predominant effect. However, the reason for the FIC result with the silver is unclear and requires further investigation but it may be due to the principal oxidation state of the silver. What is clear is that the assay used does influence the results and one assay alone should not be used to determine the overall antimicrobial efficacy of a compound.

Platinum nanoparticles have demonstrated antimicrobial efficacies against *Bacillus subtilis, Staphylococcus aureus, Pseudomonas aeruginosa* and *Escherichia coli* ^32^ whereas others have found no effect^33^. A study tested the antibacterial properties of nine different metal surfaces against *S. aureus* and *E. coli* and found that in agreement with our results, palladium demonstrated greater antimicrobial efficacy than the other metals tested^34^. A study by Kawakami *et al*. (2008) also looked at the antimicrobial efficacy of a number of metals including platinum and palladium and it was found that they were effective against *E. coli*. However, in contrast to our results, their study found that gold demonstrated little effect against *E. coli* or *S. aureus* ^35^. However, gold in nanoparticle and ionic form has been suggested to have antimicrobial activity^12^, and gold nanoparticles have also been shown to inhibit biofilm formation^36^. Although not as effective in our study, silver in other forms has demonstrated antimicrobial efficacy. Silver alginate has been shown to have antimicrobial efficacy against bacterial species isolated from burn wounds including *A. baumannii*, *K. pneumoniae* and *E. faecium* ^25, 37^. It is also well known that copper surfaces have antimicrobial efficacy against a wide range of microbes^38–40^.

The differing toxic effects of metals and their components on bacteria can be due to various mechanisms such as antioxidant depletion, deoxyribonucleic acid (DNA) damage, impaired membrane function and/or interference with nutrient assimilation^2^. The most common hypothesis for the antimicrobial action of silver involves silver ions binding to the proteins and enzymes in the cell wall, cell membrane and peptidoglycan. This causes structural changes in the cell wall, such as pits. This increases cell permeability, leading to distortion and finally lysis of the cells^40–42^. Another antimicrobial mechanism of silver is its ability to inter-chelate with the phosphorus elements in bacterial DNA, which results in impaired ability to replicate and express ribosomal subunit proteins and other cellular proteins^43–45^. Platinum’s primary cisplatin target is DNA but it also has an affinity for the sulphur and selenium donors present in many proteins^46, 47^. A palladium complex with 1,6-bis(benzimidazol-2-yl)-3,4-dithiahexane was thought to exhibit bacterial toxicity mechanisms due to metal protein binding leading to DNA damage, causing cell death. Although the chemistry of palladium is very similar to that of platinum, palladium complexes differ from those of platinum in several respects. Palladium exhibits a greater propensity to exchange ligands, which is about 105 times higher than platinum^48^. The ligand dissociation generates active metal species that can easily interact with other compounds, thus palladium complexes are toxic because of their higher reactivity^48^. The mechanistic action of gold is thought to be due to strong cationic attractions to the negatively charged plasma membrane of microbes which leads to cell membrane disruption, Reactive Oxygen Species (ROS) accumulation and consequent cell death^35, 49^. Copper ions released from copper alloys have been suggested to target bacteria by increasing ROS production and thus inducing DNA damage. However, this concept is controversial as it has further been demonstrated that disruption of the cell envelope is the mode of action of contact killing mediated by dry metallic copper surfaces^38^. In terms of our results, this concept holds true and would in part explain the low antimicrobial results when the copper was in solution. Further, there are significant differences that exist between the exposure of bacteria to copper ions and exposure to metallic copper surfaces, since the cells on dry metallic copper surfaces are not in an environment that promotes growth. Therefore, these cells face challenges that are different from those in an aqueous environment^38^. It has also been suggested that the antibacterial property of copper ions do not act like some other metals ions such as silver^50^. Thus, it may be that when the bacteria are in direct contact with copper surfaces an enhanced antimicrobial effect is achieved.

To the author’s knowledge, studies showing the antimicrobial efficacies of individual and combined metal ions has been little researched. A metal’s antimicrobial mechanistic activity is dependent on its chemical properties (for example donor atom selectivity, reduction potential), which govern their reactivity in bacterial cells. Thus, using antimicrobial agents in combination may further increase their antimicrobial efficacy by producing a synergistic effect^51–53^. In agreement with our results, work by others has also demonstrated that silver ions showed a lower MBC value for *E. coli* planktonic cells than for biofilms^54^. Since the physiology, gene expression and morphology of planktonic cells differs from those cells in biofilms, the difference in the efficacy of the antimicrobial agents against the bacteria in their different states might not be that unexpected^53–55^.

## Conclusion

In this pilot study, gold, platinum and palladium demonstrated the most effective antimicrobial activity overall for individual metal ion solutions against both planktonic or biofilm phenotypes for three pathogens and could potentially be used in biocidal formulations where intensive cleaning is required. The synergistic combinations of gold/platinum, gold/palladium and platinum/palladium have the potential to be used in a range of antimicrobial or biocidal combinations, particularly against the medically-relevant pathogens tested in this study. Other metals demonstrated some antimicrobial and synergistic activity under certain conditions and against particular cells. Overall platinum demonstrated the greatest antimicrobial efficacy. The results suggest that when used as potential antimicrobials, the combinations selected must be tested towards bacteria in their relevant physiological states. It should be noted that the results of this preliminary study were only carried out against one strain of each bacterial species and hence future work will investigate the antimicrobial effects of the metal ions against a range of bacterial isolates.

## Methods

### Cultures and Media

Stock cultures of *K. pneumoniae* strain NCTC9633 and *A. baumannii* strain 12156 were subcultured onto Nutrient agar (NA) and incubated at 37 °C for 24 h. *E. faecium* strain NCTC7171 was subcultured onto columbia blood agar (Oxoid, UK) supplemented with 5% defibrinated horse blood in a 5% CO_2_ incubator for 24 h at 37 °C. Brain heart infusion (BHIA) agar (Oxoid, UK) and brain heart infusion broth (BHIB) (Oxoid, UK) were used for all the microbiological tests for *E. faecium*. Nutrient agar and nutrient broth (NB) (Oxoid, UK) were used to perform all the assays for *K. pneumoniae* or *A. baumanii*. Gram-negative microorganisms were incubated at 37 °C overnight in an aerobic atmosphere whilst *E. faecium* was incubated in a 5% CO_2_ incubator for 24 h at 37 °C in static conditions for all the other assays in this study. All the assays were repeated at least thrice (n = 3).

### Chemical Preparation

Single element standard calibration solutions for Ion Coupled Plasma – Atomic Adsorption Spectroscopy (ICP-AAS) of 1000 mgL^−1^ of silver, copper, platinum, gold and palladium (Sigma-Aldrich, UK) were used. These were diluted with sterile water to the respective metal ion concentrations.

### Bacterial preparation

Ten millilitres of appropriate broth was put into a sterile universal for the Zone of inhibition (ZoI) assays. One hundred and fifty millilitres of appropriate broth was put into a conical flask for minimum inhibitory concentrations (MIC), minimum bactericidal concentration (MBC) and fractional inhibitory concentration (FIC) and crystal violet biofilm assays (CVBAs). These were inoculated with a single colony of bacteria and incubated overnight according to the conditions in the culture and media sub-section. Cells were harvested by centrifugation (3500 *g* for 10 min) and then washed with 10 mL sterile distilled water and vortexed to ensure an even distribution of the cell suspension. The washed cells were again re-harvested. The pellet was re-suspended in 10 mL of broth, vortexed and the resultant cell suspension was adjusted to an optical density at 540 nanometres (nm) (OD540) of 1.0 using a spectrophotometer. The cell concentration corresponded to 3.95 × 10^8^ colony-forming units per mL (CFUmL^−1^) at an OD540 of 1.0.

### Zone of inhibition assays

Respective agar was poured into sterile Petri dishes, which were then cooled and 100 μL of cell suspension was pipetted and spread across the entire area of the agar. Three equal wells (8 mm diameter) were cut out of the each agar plate using a sterile cork borer and stainless steel needle. To each of the wells, 100 μL of the metal ion solution was added. The plates were incubated as mentioned in cultures and media sub-section. The ZoI was measured using the different metal ion solution concentrations, 50 mgL^−1^, 100 mgL^−1^, 500 mgL^−1^ and 1000 mgL^−1^. Following incubation, the ZoI was measured in mm from four sides of each well to determine an average mean value (n = 12).

### Zone of inhibition assays (synergy)

For the synergy assays, the same method was carried out as above (zone of inhibition) except that two wells were cut from the agar, 6 mm apart (n = 3). The concentration of the metal ions used in the ZoI synergy assays was 50 mgL^−1^, 100 mgL^−1^, 500 mgL^−1^ and 1000 mgL^−1^. Following incubation, the inhibition zones were graded from 0 to 4, which measured as, 0–4 mm – grade 0, 4–8 mm – grade 1, 8–12 mm – grade 2, 12–1 6 mm – grade 3 and 16–20 mm – grade 4. The grade of inhibition of the metal ion solution combinations were calculated as follows;

1 $${\rm{\Sigma }}\,{\rm{metal}}\,{\rm{ion}}\,{\rm{solution}}\,{\rm{combination}}={\rm{first}}\,{\rm{metal}}\,{\rm{grade}}\,{\rm{of}}\,{\rm{inhibition}}+{\rm{second}}\,{\rm{metal}}\,{\rm{grade}}\,{\rm{of}}\,{\rm{inhibition}}$$

### Individual metal ion MIC and MBC assays

One millilitre of Triphenyl tetrazolium chloride (TTC) blue metabolic dye (Sigma-Aldrich, UK), was added into 9 mL of the OD adjusted cell suspension so that the working concentration of the dye was 0.15% w/v. To determine the MIC, 100 μL of the test samples were added to a 96 well flat-bottomed microtiter plate (MTP). One hundred microliters of bacterial suspension with the TTC dye was then added; the first column of cell/metal ion suspension was mixed, then 100 μL of the sample/bacterial mix was transferred to the column 2 wells and repeated until column 10. To column 11, 100 μL of bacterial suspension without a metal (positive control) was added and to column 12 and 100 μL of un-inoculated broth was added (negative control). After incubation, the MIC was taken as lowest concentration that inhibited the visible growth of the bacteria by comparison with the controls. Growth was indicated by a change of colour in the well to dark blue/purple. Twenty-five microliters of culture was taken from the first well that showed no growth and the last well that demonstrated growth and was pipetted onto agar plates using Miles and Misra methodology^56, 57^ (n = 3). After incubation, the lowest concentration well sample that showed no bacterial growth on the agar plate was determined to be the MBC for that test sample (n = 3).

### Fractional inhibitory concentrations assay

The bacterial suspension and test samples for the FIC test were prepared as described in the sub-section culture and media and MIC method, except that both metal ion solutions were added to the wells in a 1:1 ratio. Following incubation at 37 °C for 24 h, the FIC values were calculated as;

2 $$\begin{array}{c}{\rm{\Sigma }}\mathrm{FIC}={\rm{FIC}}\,{\rm{of}}\,{\rm{antimicrobial}}\,{\rm{A}}+{\rm{FIC}}\,{\rm{of}}\,{\rm{antimicrobial}}\,{\rm{B}}\\ \quad \quad \,\,=\,\frac{{\rm{MIC}}\,{\rm{of}}\,{\rm{antimicrobial}}\,{\rm{A}}\,{\rm{in}}\,\mathrm{combination}\,}{{\rm{MIC}}\,{\rm{of}}\,{\rm{antimicrobial}}\,{\rm{A}}\,\mathrm{alone}\,}+\frac{{\rm{MIC}}\,{\rm{of}}\,{\rm{antimicrobial}}\,{\rm{B}}\,{\rm{in}}\,{\rm{combination}}}{{\rm{MIC}}\,{\rm{of}}\,{\rm{antimicrobial}}\,{\rm{B}}\,{\rm{alone}}}\end{array}$$

depending on the FIC values, the antimicrobial interaction was evaluated as synergy = <0.5, indifference = 0.5–4.0 or antagonism = >4.0 (n = 3).

### Crystal Violet Biofilm Assay (CVBA)

#### Preparation of stainless steel coupons

Fine polished (FP) 304 grade stainless steel coupons (10 mm × 10 mm) were used in the assays. Coupons were washed thoroughly by sequentially putting the coupons into beakers each containing either acetone, methanol or ethanol (BDH, UK) for 10 min with a sterile water wash in between each, and for the final step. The washed coupons were air dried and stored in sealed plastic containers at room temperature until used.

#### Biofilm formation and CVBA (adapted from Christen *et al*. 1985)

The cell suspension was prepared in the same manner as described in culture and media sub-section. Twelve well culture plates were used to grow the biofilms. Cleaned coupons were placed in the centre of the well with the fine polished surface facing upward. One millilitre of adjusted OD cell suspension was added to each well and incubated for 7 days at 37 °C to produce a biofilm. Plates were wrapped in Parafilm^TM^ to prevent moisture loss and air contaminants over the long incubation time. After incubation, the stainless-steel coupons were carefully washed with 2 mL of sterile distilled water using a pipette to remove any loose planktonic cells whilst avoiding damaging the biofilms. The coupons were air dried at room temperature for 2 h. One millilitre of metal ion solution at 500 mgL^−1^ was added into each respective well. Respective agar broths were also added into one of the wells to serve as a negative control. The plates were incubated for 24 h at 37 °C. Following incubation, the metal ion solutions were removed. The coupons were carefully washed with 1 mL of sterile distilled water and were air dried at room temperature. One millilitre of 0.03% crystal violet solution (Oxoid, UK) was added into each well with a coupon and left for 30 min. The coupons were gently washed with 2 mL sterile distilled water to remove any excess stain. The coupons were placed into new 12 well plates and air dried at room temperature for 1 h. One millilitre of 33% glacial acetic acid (BDH, UK) was added to each well and left for 30 min to solubilise any stained biofilm. The solution was removed and the absorbance measured at OD590 (n = 3). Bacterial biofilms were divided into breakpoint categories; OD < 0.067 antimicrobial; OD ≥ 0.068 but ≤ 0.135 partial antimicrobial activity; ≥OD 0.136–≤0.270 negligible antimicrobial activity; >0.271 no antimicrobial activity. These values were determined using quartiles of the lowest OD value determined from the control.

### Statistical analyses

Mean values were used to compare the antimicrobial efficacy results of the metal ion solution samples at varying concentrations. Standard deviation or standard error were calculated to analyse the distributions of the data from the mean value, and confidence intervals of 95% were calculated for the ZoI, FIC and MBC synergy tests results to plot error bars.
